# Communicating the Benefits and Harms of Colorectal Cancer Screening Needed for an Informed Choice: A Systematic Evaluation of Leaflets and Booklets

**DOI:** 10.1371/journal.pone.0107575

**Published:** 2014-09-12

**Authors:** Maren Dreier, Birgit Borutta, Gabriele Seidel, Inga Münch, Silke Kramer, Jürgen Töppich, Marie-Luise Dierks, Ulla Walter

**Affiliations:** 1 Institute for Epidemiology, Social Medicine and Health Systems Research, Hannover Medical School, Hannover, Germany; 2 Department 2 Effectivity and Efficiency of Health Education, Federal Centre for Health Education (BZgA), Köln, Germany; Sapporo Medical University, Japan

## Abstract

**Objective:**

Evidence-based health information (EBHI) can support informed choice regarding whether or not to attend colorectal cancer (CRC) screening. The present study aimed to assess if German leaflets and booklets appropriately inform consumers on the benefits and harms of CRC screening.

**Methods:**

A systematic search for print media on CRC screening was performed via email enquiry and internet search. The identified documents were assessed for the presence and correctness of information on benefits and harms by two reviewers independently using a comprehensive list of criteria.

**Results:**

Many of the 28 leaflets and 13 booklets identified presented unbalanced information on the benefits and harms of CRC screening: one-third did not provide any information on harms. Numeracy information was often lacking. Ten cross-language examples of common misinterpretations or basically false and misleading information were identified.

**Discussion:**

Most of the CRC screening leaflets and booklets in Germany do not meet current EBHI standards. After the study, the publishers of the information materials were provided feedback, including a discussion of our findings. The results can be used to revise existing information materials or to develop new materials that provide correct, balanced, quantified, understandable and unbiased information on CRC screening.

## Introduction

In the past, cancer screening procedures gained increasing significance for public health, and were promoted in many countries [1]. However, in recent years, it became clear that the benefits of some procedures may have been overemphasised and their harms underemphasised [2]. Potential harms of cancer screening include adverse effects from false-positive test results (mental stress, diagnostic evaluation), from the procedure itself, and from overdiagnosis [3]–[5]. As screening addresses healthy people who have a very small risk of ever developing the target disease, even very rare potential harms of the screening procedure are relevant. Thus, the individual should have the possibility to make an informed choice for or against the screening test [6], [7]. In Germany, statutory health insurance covers colorectal cancer (CRC) screening tests, including the Guaiac-based faecal occult blood test (gFOBT) and screening colonoscopy, for persons aged 50 years and older, comprising about 85% of the population. In contrast to the FOBT, the colonoscopy is more a preventive than an early diagnostic screening test because endoscopic measures allow the detection and removal of CRC precursors (polyps, adenoma) [8]. As colonoscopy is an invasive procedure [9], [10], special efforts are needed to promote informed decision-making. The informed choice to attend or not to attend CRC screening and which test to choose may be supported with appropriate evidence-based health information (EBHI) materials [6], [11]. EBHI requires balanced, unbiased, quantified, understandable, and evidence-based information about CRC and both the benefits and harms of CRC screening [12], [13].

To estimate the extent of benefits and harms, numerical figures are essential. It is important to present the statistics in a comprehensible and unbiased manner [12], [14], [15]. To begin with, probabilities should be presented as natural frequencies [16], [17] because natural proportions are easier to understand than percentages (e.g. 80 out of 100 instead of 80%). A reference number (denominator) is needed to estimate the magnitude of effects. If possible, the same denominator should be used to ensure the comparability of the numbers. Regarding information on “lifetime” measures, the following messages are recommended: 1. When discussing risks, limited time frames (e.g. “in the next 10 years”) are easier to understand than “lifetime” figures [14]. 2. When comparing the benefits of having a given screening test, use of the amount of absolute risk reduction (ARR) is preferred because relative risk reduction (RRR) tends to overestimate the effect [16]–[18]. For example: If five out of 1,000 screening participants develop CRC compared to 25 out 1,000 without screening, RRR = 80% (or: of 25 persons, only 5 out of 1,000 develop CRC = 80% less). In contrast, ARR = 20 out of 1,000 or 2%. This means that 20 out of 1,000 screening participants benefit from screening and do not develop the disease, compared to those not participating in screening. The temporal dimension of the risk reductions should also be indicated (for example: “1,000 people have an annually FOBT over 10 years”). Otherwise, essential information for benefit estimation would be lacking.

In Germany, information about CRC screening is provided by different healthcare stakeholders, including governmental organisations, foundations, healthcare providers, and health insurance companies. Some of the existing information materials do not meet EBHI standards, but rather focuses on the benefits of screening and/or strongly encourages participation in screening [19]. In the context of the National Cancer Plan [20], the Federal Ministry of Health funded a CRC screening project to identify information materials that meet EBHI standards. We developed a list of criteria to assess whether print media like leaflets and booklets provide reliable, correct, understandable and unbiased information on CRC screening (gFOBT and colonoscopy) [21]. This article reports whether and what kind of information on benefits and harms of CRC screening is contained in such leaflets and booklets in Germany. The aim is explicit not to discuss, which screening test to choose or to assess CRC screening strategies in Germany.

## Materials and Methods

### Ethics statement

The study protocol was approved by the ethics committee of Hannover Medical School (Application No. 1803–2013).

The study methods included the following steps: 1) development of an assessment tool for expert use [21], 2) identification of consumer information materials on CRC screening, 3) assessment of the identified materials from an expert's view, and 4) assessment of the materials from the consumer's view. This paper reports the results from 2) and 3).

### Identification of leaflets and booklets on CRC screening

In August 2010, stakeholders such as governmental organisations, foundations, healthcare providers, statutory health insurance companies, medical and scientific societies, and professional associations in Germany (excluding the pharmaceutical industry) were systematically identified and asked via email for leaflets and booklets on CRC screening. Information materials from the pharmaceutical industry were excluded due to potential conflicts of interest. A reminder email was sent two weeks later, if necessary. The websites of these institutions were also searched. The latest versions of available print media and online printable documents (PDF or MS Word format) on CRC screening (in German) addressing persons with an average risk of CRC were included in the analysis. Eligible materials had to provide information at least on the two CRC screening methods reimbursed by German statutory health insurances: gFOBT and colonoscopy. Information materials designed only to help persons determine their individual CRC risk, regional print media, or leaflets and booklets targeting CRC patients or people with an increased risk of CRC (e.g. persons with inflammatory bowel disease, familial adenomatous polyposis, hereditary non-polyposis colorectal cancer, or increased incidence of CRC in first- or second-degree relatives) were excluded. The provision of additional information on treatment options was no reason for exclusion.

### Assessment of the identified health information

The information materials were assessed independently by two out of four reviewers (MD, BB, GS, IM) using an extensive list of 230 criteria [21] designed to evaluate information on the gFOBT and colonoscopy. Any discrepancies between the reviewers were solved by consens, if necessary, including a third person. The list is divided into four domains: A. Content issues, B. Formal issues, C. Presentation and understandability and D. Neutrality and balance (Table S1), representing a maximum of possible information. Not all of the criteria are essential for high-quality information. A and B criteria are rated multi-dimensionally, i.e. on reporting, correctness, presentation and evidence level. For each item an accompanying text input was possible that helps to make the reviewers' decisions transparent. C criteria were rated with “yes”, “rather yes”, “rather no”, “no”, “not applicable”. A manual supported the assessment by giving the correct answers based on systematic reviews, HTA reports, and guidelines. To illustrate examples for false or misleading information, these examples were translated into English language and reviewed by a certified translator who is a native English speaker. The original German wordings are provided in the supplement. Differences between leaflets and booklets were restricted to qualitative comparisons because the cell count was too small for sound statistical analysis.

#### Baseline risks

Baseline risks, namely the risk of developing the target disease and dying from it, give an impression of the disease severity. These risks are derived from incidence and mortality rates (frequencies of getting or dying from a disease). The corresponding criteria on the list gather information on the precursors, incidence and mortality of CRC, including age- and sex-specific rates, comparisons with risks associated with other cancers, everyday risks, and the natural course of disease (Table 1).

**Table 1:** Information on baseline risks.

Colorectal cancer information	Leaflets (n = 28)				Booklets (n = 13)			
	Total		False information		Total		False information	
	n (%)		n (%)		n (%)		n (%)	
Meaning of precursors/ polyps is stated	22	(79%)	1	(4%)	10	(77%)	1	(8%)
Frequency of adenomas is stated	5	(18%)	3	(11%)	2	(15%)	0	(0%)
Incidence is stated	16	(57%)	6	(21%)	9	(69%)	3	(23%)
Incidence by sex is stated	3	(11%)	0	(0%)	5	(38%)	2	(15%)
Incidence bye age is stated	2	(7%)	0	(0%)	1	(8%)	1	(8%)
Mortality is stated	18	(64%)	0	(0%)	6	(46%)	0	(0%)
Mortality by sex is stated	1	(4%)	0	(0%)	2	(15%	2	(15%)
Mortality by age is stated	1	(4%)	0	(0%)	0	(0%)	0	(0%)
Remaining lifetime risk of developing CRC is stated	0	(0%)	0	(0%)	0	(0%)	0	(0%)
Remaining lifetime risk of dying from CRC is stated	0	(0%)	0	(0%)	0	(0%)	0	(0%)
The age-related risk of developing CRC within a period of time is stated	0	(0%)	0	(0%)	1	(8%)	0	(0%)
The age-related risk of dying within a period of time is stated	2	(7%)	0	(0%)	2	(15%)	0	(0%)
The risk of developing CRC compared to other risks is stated	11	(39%)	0	(0%)	10	(77%)	0	(0%)
The risk of developing CRC compared to other types of cancer is stated	0	(0%)	0	(0%)	0	(0%)	0	(0%)
The risk of dying from CRC compared to other risks is stated	6	(21%)	0	(0%)	7	(54%)	0	(0%)
The risk of dying from CRC compared to other risks is stated	4	(14%)	1	(4%)	1	(8%)	0	(0%)
The natural progression of the disease is stated	6	(21%)	0	(0%)	1	(8%)	0	(0%)

#### Benefits

The following parameters were included to assess the potential benefits of CRC screening:

1. CRC-incidence
2. CRC-mortality
3. All-cause-mortality.

They were assessed with respect to the benefits of CRC screening in general, and to the benefits of colonoscopy and the FOBT in particular (Table 2). Both qualitative and quantitative information (e.g. “screening prevents deaths from CRC” and “screening prevents death from CRC in 3 out of 1000 cases”, respectively) was collected. Quantitative information was further divided into ARR, RRR and the number needed to screen (NNS), which is calculated as the reciprocal value of the ARR.

**Table 2:** Ten examples of commonly or basically false and misleading information in CRC screening leaflets and booklets.

No.	Examples of false information	Explanation
**Baseline risks**		
1	“Many of the benign lesions become cancerous.”	The actual risk of a polyp becoming malignant is ≤15% depending on the size of the polyp.
		(Only the minority of polyps develop into cancer, while CRC usually arises from polyps)
2	“Nearly half of all CRC patients die of the disease each year.”; „.. more than 50% of all persons affected, that is about 30.000 individuals, die of CRC each year.”	Annual number of deaths referred to annual new cases is misinterpreted as case fatality rate, resulting in large overestimation of the risk of death.
		(Case fatality rate calculated from annual number of deaths and new cases)
3	“Each year, there are about 69.000 new cases and 27,000 deaths from CRC.”	Statements mentioning annual CRC cases and deaths in one sentence may be misunderstood as implying that about one-third of all cases will die within the first year, resulting in overestimation of the risk of death.
		(Case fatality rate calculated from annual number of deaths and new cases)
**Benefits of screening**		
4	“Prevention has benefit in terms of years of life and quality of life in all cases.”	In actuality, prevention only benefits those, whose CRC is detected earlier or is prevented by screening resulting in a longer life. This is only true for a small minority of patients (annual CRC incidence is about 100-500 per 100.000 persons, depending on age group). The vast majority will not have any benefit as they would never develop CRC.
		(Real benefit only for those who will actually develop *and* die from CRC)
5	“Early diagnosis. You should talk to your doctor if: – you detect blood in your stool – your stool has changed – you have unexplained abdominal pain.”	Mixing of screening (that addresses people without symptoms) and diagnostic procedures (that addresses people with symptoms) may increase widespread false understanding about screening, especially in screening-non-adherent persons who justify their non-participation with lack of symptoms.
	A celebrity saying: “The first time I went to colorectal cancer screening, it was because I had symptom X.”	(diagnostic vs. screening procedures)
**Harms of screening**		
6	“harmless drug preparation”	Possible adverse effects include cardiovascular symptoms, allergies, nausea, cramps and pain.
		(Preparation for the procedure also has side effects)
7	“all preliminary and early stages can be removed completely”	Larger polyps are usually removed in a second examination.
8	“.. can be removed without risk.” (polyps)	Typical risks include bleeding and perforation.
**Test accuracy**		
9	Colonoscopy “is the safest way to prevent CRC..” or “provides the highest safety”	“Safe”, “safest” or “safety” may be misinterpreted as referring to adverse effects instead of test sensitivity.
	(10/28 leaflets, 1/13 booklets)	(Sensitivity vs. risks)
10	“A negative stool test (without findings) means a further residual risk of 70 to 80%.”	This suggests that someone with a negative stool test has a high risk of CRC, but the actual risk is very low.
		(False-negatives vs. negative predictive value)

#### Harms

The potential harms of CRC screening were examined with reference to screening colonoscopy and FOBT. Three main areas (modified from [3]) were considered:

1. Harms of the screening test including those due to preparation, the procedure itself, and accompanying measures (anaesthesia/sedation)
2. Risks of inaccurate test results, including false-positive results (that may lead to complications of follow-up, emotional distress, and lost work days) and false-negative results (that miss disease and thus cause a delay in treatment)
3. Harms of overdiagnosis from true-positive identification of CRC or precancerous abnormalities (that would not have been harmful and led to death [3], [4], [22])

#### Numerical data

Numerical data in the leaflets and booklets were assigned to the corresponding criteria. Free text was added, citing the wording and information on whether the number was presented as a natural frequency, with a denominator and time frame. In addition, domain C provided aggregated criteria on the presentation of numerical data (e.g. the use of natural frequencies instead of percentages, reference values, and same denominators).

## Results

Seventy-two of the 142 stakeholders identified (50.7%) responded. In combination with the internet search, a total of 71 information materials were identified, 41 of which (28 leaflets and 13 booklets) met the inclusion criteria. A detailed list of the included materials is provided in the supplement (Table S2). The majority of leaflets were published by non-profit organisations such as foundations (17/28) and state ministries (6/28), while the booklets were mainly published by scientific societies (6/13).

### Information on baseline risks

Four of five materials explained the meaning of precursors for the development of CRC, but information on the frequency of these polyps or adenomas was clearly less often given (Table 1). We identified some false statements suggesting, for example, that *many* of the precursors become cancerous, though this is only true in 5–15% of cases (Table 2, Example 1) [10], [23]. Information on CRC incidence was presented in 61% of the leaflets (17/28) and 85% of the booklets (11/13), but mainly without stratification by sex and age. Information on CRC mortality was included in nearly two-thirds of the leaflets (18/28) and half of the booklets (6/13). Information on the remaining lifetime risk was not given and only two leaflets and two booklets mentioned age-related risks of developing CRC and/or dying from CRC. More frequently, the information materials compared CRC risks to other risks. In most cases, this was done by describing CRC as the second most common cause of developing or dying from cancer out of all types of cancerous diseases. Only a minority of materials described the natural progression of the disease.

In 11/28 leaflets and 4/13 booklets, we identified a common source of misleading information, which arises from describing CRC incidence and mortality of CRC in a single sentence (Table 2, Example 3). As a result, the reader may get the impression that about one-third of the newly diseased will die within one year and thus will overestimate the case fatality rate of the disease. This example also shows that we accepted the absolute number of newly diseased and the absolute number of cases of death as incidence and mortality even though the correct number should include a reference population as a denominator. Further results on the rating of numerical data are described in detail below.

### Information on the benefits of CRC screening

Most of the leaflets and booklets described *general* benefits of CRC screening in terms of decreasing the incidence and mortality of CRC, for example: “cancer can be prevented”, “cancer is curable if detected early”, “screening reduces deaths caused by CRC” (Table 3). In contrast, the *specific* benefits of a given screening test were clearly less frequently mentioned. While about one-third of the booklets mainly indicated the positive effect of the FOBT or colonoscopy on the CRC-mortality, leaflets rather focused on the reduction of CRC incidence by colonoscopy.

**Table 3:** Reported benefits of CRC screening in general and for colonoscopy and the FOBT in particular in the identified leaflets (n = 28) and booklets (n = 13).

Benefits	Leaflets (n = 28)				Booklets (n = 13)			
	Total		False information		Total		False information	
	n (%)		n (%)		n (%)		n (%)	
**Benefits of CRC screening in general**								
Reduction of CRC incidence	24	(86%)	1	(4%)	10	(77%)	0	(0%)
Reduction of CRC mortality	26	(93%)	1	(4%)	12	(92%)	0	(0%)
Reduction of total mortality	0	(0%)	0	(0%)	0	(0%)	0	(0%)
**Benefits of colonoscopy**								
Reduction of CRC incidence	4	(14%)	0	(0%)	1	(8%)	0	(0%)
Reduction of CRC mortality	1	(4%)	0	(0%)	4	(31%)	0	(0%)
Reduction of total mortality	1	(4%)	0	(0%)	0	(0%)	0	(0%)
**Benefits of the FOBT**								
Reduction of CRC incidence	1	(4%)	1	(4%)	0	(0%)	0	(0%)
Reduction of CRC mortality	2	(7%)	0	(0%)	3	(23%)	0	(0%)
Reduction of total mortality	0	(0%)	0	(0%)	0	(0%)	0	(0%)

FOBT: faecal occult blood test, guaiac-based.

CRC: colorectal cancer.

False messages were rare. Statements, such as “cancer disease that can be prevented in 100% of cases” were rated as false. Table 2 shows further important examples for basically false and misleading information on screening benefits. Example 4 claims that it is an advantage for everyone to attend screening, neglecting to state that screening is only useful for individuals who actually develop CRC. Example 5 is also misleading because it mixes information about screening versus diagnostic examinations, which may increase widespread false beliefs about screening, especially in those who refuse screening because they have no symptoms.

### Information on the harms of CRC screening

#### Harms attributed to the screening procedure

Unlike colonoscopy, there are no direct adverse effects attributed to the FOBT test. Those associated with colonoscopy are divided into harms that occur during colonoscopy preparation, sedation, and the colonoscopy procedure itself (Table 4). Only 3 out of 41 (7%) information materials indicated any harms due to the preparation or sedation. Harms of the colonoscopy procedure itself were reported more frequently. Half of the leaflets and nearly two-thirds of the booklets gave information on common risks, for example, by characterising colonoscopy as being a “low-risk” procedure or having “few complications”, and causing possible pain. Information on further adverse effects like bleeding, infection, and perforations was included less often. Only one leaflet and two booklets indicated the risk of death. More than one-third of the leaflets (10/28) and one-fifth of the booklets (2/11) did not mention harms at all.

**Table 4:** Reported harms of screening colonoscopy due to colonoscopy preparation, sedation and/or the procedure itself in the identified leaflets (n = 28) and booklets (n = 13).

Risks	Leaflets (n = 28)				Booklets (n = 13)			
	Total		False information		Total		False information	
	n (%)		n (%)		n (%)		n (%)	
**Preparation**								
Common risks	1	(4%)	1	(4%)	1	(8%)	0	(0%)
Cardiovascular symptoms	0	(0%)	0	(0%)	2	(15%)	0	(0%)
Nausea	0	(0%)	0	(0%)	1	(8%)	0	(0%)
Allergies	0	(0%)	0	(0%)	0	(0%)	0	(0%)
Cramps	0	(0%)	0	(0%)	1	(8%)	0	(0%)
Pain	0	(0%)	0	(0%)	1	(8%)	0	(0%)
**Sedation**								
Common risks	0	(0%)	0	(0%)	0	(0%)	0	(0%)
Affected respiratory/ respiratory arrest	0	(0%)	0	(0%)	3	(23%)	0	(0%)
Cardiovascular symptoms	0	(0%)	0	(0%)	1	(8%)	0	(0%)
Nausea	0	(0%)	0	(0%)	0	(0%)	0	(0%)
**Procedure**								
Common risks	14	(50%)	2	(7%)	8	(62%	2	(15%)
Pain	14	(50%)	11	(39%)	8	(62%)	1	(8%)
Cardiovascular symptoms	0	(0%)	0	(0%)	0	(0%)	0	(0%)
Nausea	0	(0%)	0	(0%)	0	(0%)	0	(0%)
Bleeding	2	(7%)	0	(0%)	4	(31%)	1	(8%)
Infections	3	(11%)	0	(0%)	2	(15%)	0	(0%)
Perforations	2	(7%)	0	(0%)	3	(23%)	1	(8%)
Death	1	(4%)	0	(0%)	2	(15%)	0	(0%)

In some cases (especially leaflets) the risk reporting was rated as being incorrect. Most frequently, the colonoscopy procedure was falsely characterised as being “pain-free” or “non-painful”. Common statements describing colonoscopy as being “without any problems” or “safe” were likewise considered false. Some materials were rated as having excessive or very low estimates of the frequencies of possible adverse effects. For further examples, see Table 2, No. 6–8.

#### Harms attributed to inaccurate tests

Statements on the quality of the screening tests were assessed (Table 5). About two-thirds of the leaflets and booklets provided general information on the test accuracy of colonoscopy. However, this information was misleading in half of the leaflets, as shown in Table 2, Example 9: Colonoscopy was sometimes described as being “ the safest (German: sicherste) way to prevent CRC” or as “providing the highest safety (German: Sicherheit)”. This obviously refers to the high sensitivity and specificity of the colonoscopy in the sense of being “reliable”, but may be misinterpreted as meaning that the procedure is completely safe and without any possible adverse effects.

**Table 5:** Reported accuracy of CRC screening tests in leaflets (n = 28) and booklets (n = 13).

Test accuracy	Leaflets (n = 28)				Booklets (n = 13)			
	Total		False information		Total		False information	
	n (%)		n (%)		n (%)		n (%)	
**Colonoscopy**								
General statements about test accuracy are made	20	(71%)	0	(0%)	8	(62%)	0	(0%)
Sensitivity is stated	2	(7%)	0	(0%)	4	(31%)	0	(0%)
Specificity is stated	0	(0%)	0	(0%)	0	(0%)	0	(0%)
Frequency of false-positive results is stated	0	(0%)	0	(0%)	1	(8%)	0	(0%)
Frequency of false-negative test results is stated	0	(0%)	0	(0%)	0	(0%)	0	(0%)
Predictive values:								
Positive predictive value/frequency of correct-positive results is stated	1	(4%)	0	(0%)	1	(8%	0	(0%)
Negative predictive value/correct-negative results is stated	0	(0%)	0	(0%)	1	(8%)	0	(0%)
**FOBT**								
General statements about test accuracy are made	9	(32%)	0	(0%)	8	(62%)	0	(0%)
Sensitivity is stated	9	(32%)	1	(4%)	3	(23%)	0	(0%)
Specificity is stated	0	(0%)	0	(0%)	0	(0%)	0	(0%)
Frequency of false-positive results in patients without CRC is stated	1	(4%)	0	(0%)	4	(31%)	1	(8%)
Frequency of false-negative test results in patients with CRC is stated	0	(0%)	0	(0%)	3	(23%)	0	(0%)
**Predictive values:**								
Positive predictive value/frequency of correct-positive results is stated	2	(7%)	0	(0%)	2	(15%)	0	(0%)
Negative predictive value/correct-negative results is stated	8	(29%)	0	(0%)	7	(54%)	1	(8%)

As for the FOBT, general information on test accuracy was given in one-third of the leaflets and in nearly two-thirds of the booklets, often in comparing the FOBT to colonoscopy as “less reliable” or “not sufficiently reliable”. Accuracy criteria for the FOBT test were indicated more often than those for colonoscopy, especially sensitivity and negative predictive value data. Table 2, Example 10 shows a false and very misleading description of false-negatives, which are presented as negative predictive values, giving the reader the impression, that someone with a negative FOBT has a high risk of CRC.

Regarding the information on test properties, predictive values give consumers the best information because they display the actual proportions of correct or false results out of all positive or negative results. Thus, these values represent the consumers' view and qualify as patient-oriented information. However, such information was provided for the FOBT in only about half of the identified materials, and even less often for the colonoscopy.

#### Harms attributed to overdiagnosis

No information on overdiagnosis was found in any of the leaflets and booklets.

### Numerical data

**Figure 1:**
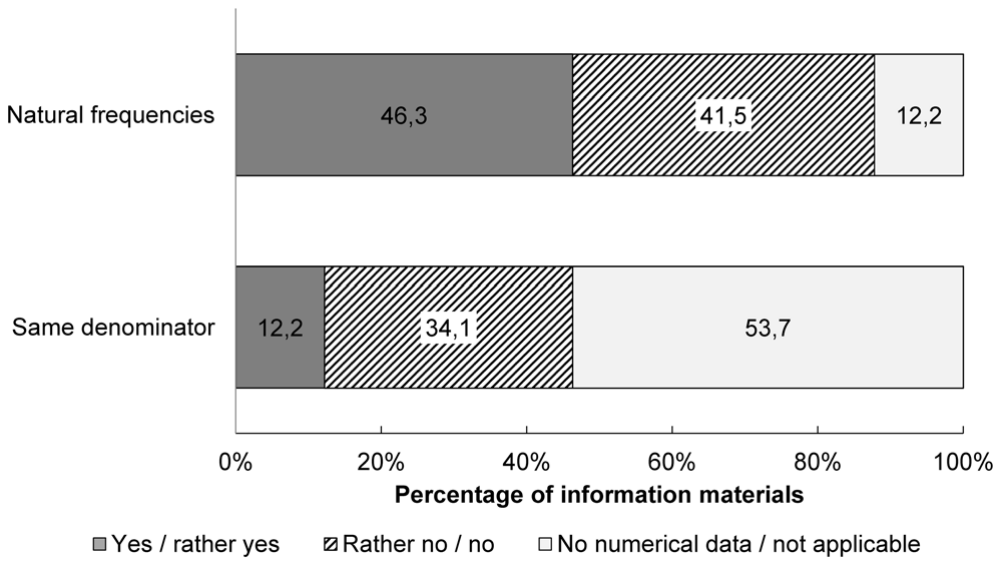
Presentation of numerical data (28 leaflets, 13 booklets). Results from the criteria characterising the quality of numerical data: 1. Natural frequencies instead of percentages are used. 2. Same denominators are used.

Overall, about half of the leaflets and booklets presented numerical data as natural frequencies, and one quarter of these materials used the same denominator within one subject (Figure 1).

More detailed analysis revealed that most of the information materials (21/28 leaflets, 12/13 booklets) included some quantification of baseline risks in terms of incidence and mortality. However, these numbers were mainly presented as absolute numbers of cases in Germany without a reference population and without a proportion such as 100 out of 100,000.

Only some of the leaflets and booklets provided quantitative information on benefits and harms. Regarding colonoscopy, three leaflets and one booklet describing the RRR for *CRC incidence* reported, for example, that “80% of all CRC is preventable”. We raise two points of criticism against such statements. First, presenting numerical data as natural frequencies (e.g. 80 out of 100) is considered more comprehensible than percentages [12], [16]. Second, failing to indicate the ARR along with the RRR leads to an enormous exaggeration of the benefit [14], [17], [18].

The positive effects of colonoscopy on the *CRC mortality* were quantified in six information materials (15%), four of which correctly indicated, in accordance with the current evidence base, that the extent of benefit is currently unknown. The other two only described the RRR without giving the ARR (for example: “according to experts, early colonoscopy could save more than three-quarters of …”). Regarding the FOBT, estimates of the reduction of mortality are available from meta-analysis of randomised controlled trials, providing the highest level of evidence [24]. These figures were found in less than 10% of the identified information materials (3/41), most of which described the amount of ARR rather than RRR (for example: “1 to 3 out of 1,000 persons less die with screening than without screening”). One material reported the number needed to screen (NNS) to prevent one case of death caused by CRC.

Information on the harms of the screening colonoscopy seldom included numerical data. Quantitative information on the following was provided in leaflets versus booklets: common adverse effects (3/28 vs. 4/13), bleedings (1/28 vs. 3/13), perforations (2/28 vs. 3/13), and mortality (1/28 vs. 1/13). Most of these numbers were comprehensibly presented as natural frequencies.

### Balance of benefits and harms

**Figure 2:**
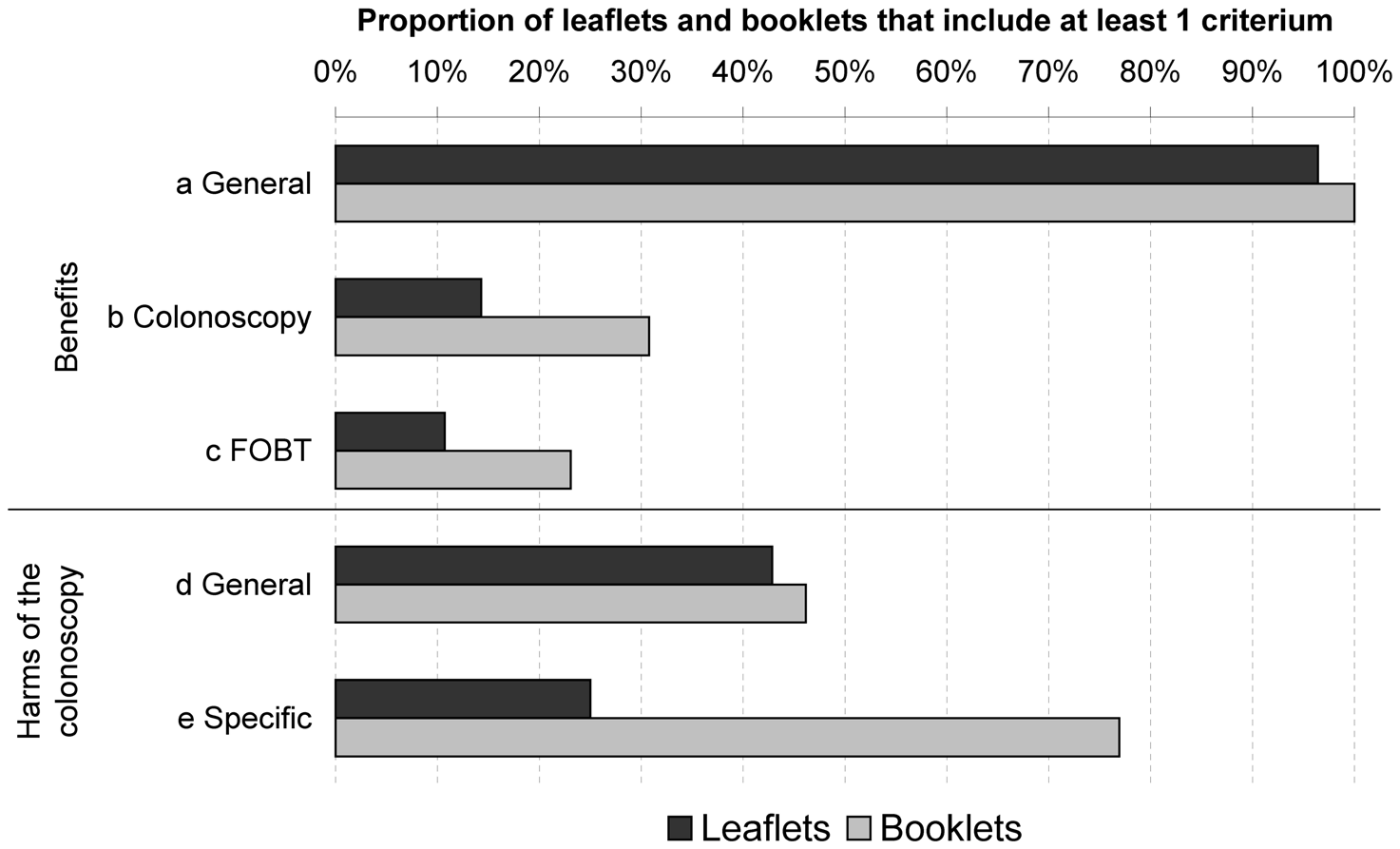
Aggregated results for reported benefits and harms, stratified by leaflets and booklets. The figure indicates whether a leaflet contains any information on the benefits of CRC screening a) in general or specifically for b) colonoscopy or c) the FOBT, and whether it contains any information on the d) general and e) specific harms of colonoscopy. To be rated positive for harms, it was not sufficient if the only information about possible harms referred to pain, stating incorrectly that there is no pain involved.

Whereas almost all leaflets and booklets provided information on the general benefits of CRC screening, only about half of the leaflets and booklets described the general harms of the colonoscopy (Figure 2). The reverse is true for specific benefits and harms, whereby specific harms were presented more often than specific benefits in the case of both colonoscopy and FOBT. Overall, specific information was more often provided in booklets than in leaflets.

## Discussion

Thirteen German booklets and 28 leaflets on CRC screening were identified. Their content and correctness of reporting the benefits and harms of CRC screening was assessed using a comprehensive list of criteria. We found that the standards of evidence-based health information were met only partially. First, information on benefits and harms was presented in an unbalanced manner, generally in favour of the benefits; however specific harms were reported more often than specific benefits. The vast majority of the materials provided at least qualitative information on potential benefits, whereas nearly one-third of the materials did not mention potential harms associated with CRC screening at all. Quantitative information about benefits and harms was mostly lacking. The numerical data presentations showed deficits in understandability and were partly misleading in favour of screening. Some materials contained false and/or distorted information.

We used a two-way approach to searching for leaflets and booklets: written email enquiries, and systematic searches of the websites of a broad range of stakeholders. Fifty percent of the stakeholders responded, which might be because not all of them provide information materials on CRC screening. As we additionally searched the websites, we are rather confident that we found almost all leaflets and booklets that met the inclusion criteria. The reviewers were not blinded to the identity of producers of the information materials. Thus, ratings could be biased in favour of the leaflets and booklets from scientific organisations, but this was probably rare, as false or misleading information was found in nearly all of the leaflets and booklets. As materials provided by the pharmaceutical industry were excluded, our overview of the quality of leaflets and booklets might not be representative of all CRC screening leaflets and booklets in Germany. This might be especially true for certain screening tests (e.g. immunological stool tests) that are not reimbursed by statutory health insurance companies. Despite the extremely high number of criteria assessed, our list was not entirely complete. First, we focused on traditional outcomes and did not use quality of life (QoL) to characterise the benefits of CRC screening in supplement to CRC incidence and mortality data [3]. However, QoL was partially taken into account in the context of describing adverse effects of the screening procedure. Thus, QoL criteria will be added to the list as parameters of patient-related outcomes [3]. Second, as we focused on tests reimbursed by German statutory health insurance companies, flexible sigmoidoscopy was not included though there is highest-level evidence indication that it reduces CRC incidence and mortality [25]. However, our aim was to assess the information materials; to evaluate the current screening strategies in Germany is of high importance but would go beyond the scope of this study. Third, we collected information on the quality of the screening tests but not on the consequences of false-positive or -negative test results, including physical or psychosocial harms from the test results as well as unnecessary follow-up procedures [26]. These consequences will be acknowledged in detail in future information material assessments.

A quality assessment that goes beyond previous tools by considering the correctness of information was used in the present study. To our knowledge, this study on the quality of written information materials provides the most thorough and in-depth evaluation of reported benefits and harms of CRC screening available. The use of a comprehensive list of criteria enabled us to detect missing information in detail. However, it is still unclear which information should be included in either brief or extensive information materials because the criteria list reflects the maximum content requirements. The main strength of this approach, apart from its comprehensiveness, is its ability to rate the correctness of information. Previous tools, which focus on criteria that characterise structural and process quality as surrogate parameters for content quality, might not always give true results [27]. Other tools for the assessment of content quality [28] do not assess the correctness of information and might even rate false information as being of good quality. The amount of false information detected in the leaflets and booklets underscores the need to directly assess the correctness of such information. Ten examples of common misinterpretations or basically false and misleading information were presented in this paper. Although these examples originate from materials written in German, they have cross-language validity and thus might be very helpful for health information providers worldwide. For example, when searching the National Cancer Institute's website for information about colorectal cancer screening [29], we found the simultaneous reporting of CRC incidence and mortality data in one sentence. In the present study, we showed that this may lead to exaggeration of the anticipated CRC case fatality rate (Table 2, Example 3).

Many of the leaflets and booklets must be revised to comply with EBHI standards. Thus, most of the information materials do not meet the prerequisites for informed choice. The identified deficits clearly indicate that the principles of good communication practice have not been adopted by the producers of information leaflets and booklets on CRC screening [6]. Obviously, the effects of promotional media campaigns that focus on the benefits of CRC screening are still predominant, while the new political strategy of promoting informed choice in an unbiased manner has caught these consumer information producers unprepared. Besides, people might be afraid that an informed choice will decrease participation in screening. High participation rates are necessary to support the quality of screening programs and to be able to measure benefits on a population level. From an ethical point of view, the informed choice is prior to the aim of increasing the uptake [13]. However, the effect of informed-decision making in screening remains unclear as studies found either increased, or decreased attendance, or no effects [30]–[34].

There is an urgent need to address the identified shortcomings. As a first step, the results of this study were already presented to different providers of the evaluated leaflets and booklets at a workshop organised by the German Federal Centre for Health Education (BZgA) to. In addition, individual rating results were submitted to the providers in writing. The public may not be prepared for EBHI that includes information on harms as well as numerical data or statements regarding the uncertainties of research results [35]. Moreover, not all people are capable of making an informed choice. Therefore, special effort is needed to develop information materials for people with different levels of health literacy [11], . Further effort is needed to improve and implement current knowledge about the production of evidence-based health information and to establish minimum content requirements to enable informed choices.

EBHI on benefits and harms may support an informed decision making [32], [33] regarding whether or not to attend CRC screening [2], [6]. Our list of criteria examines whether health information meet EBHI standards, but cannot directly assess whether it is sufficient to support informed choice [21]. The criteria list is used together with a manual that provides correct answers in order to minimise subjectivity and to facilitate the rating process. Challenges of the manual include the need for large time resources for regular updates to incorporate the latest evidence, as well as conflicts arising from different interpretations of the current evidence, for example, when experts disagree on the actual numbers characterising the benefits and harms in a leaflet on breast cancer screening [37]. The criteria list might also be used to revise existing information materials or to produce new ones. The rating principle should be adapted when assessing screening information for other tests or types of cancer.

## Supporting Information

Table S1 (DOC)

Table S2 (DOC)
