# Supplementary material for: Communicating the Benefits and Harms of Colorectal Cancer Screening Needed for an Informed Choice: A Systematic Evaluation of Leaflets and Booklets
Source: PLoS One. 2014 Sep 12;9(9):e107575. doi: 10.1371/journal.pone.0107575 (PMC4162645; doi:10.1371/journal.pone.0107575)
Supplement: Table S1 — (DOC) [file pone.0107575.s001.doc]

Table S1: Content structure of the list of criteria for evaluating consumer information materials on colorectal cancer (CRC) screening (n=230 criteria*)

| **Domain (n criteria)** | **Category (n criteria)** | **Subtopic (n criteria)** | **Dimensions to rate** |
| --- | --- | --- | --- |
| **Specific** |  |  |  |
| **A. Content issues (130)** | Information on CRC and CRC screening (32) | CRC screening (12) | Reported: yes / no |
|  |  | Aetiology and epidemiology of colorectal cancer (German data) (20) | Correct: yes / no / unclear |
|  |  |  | Presentation format: text / number / chart / Table / figure |
|  |  |  | Evidence level reported: yes / no / lack of evidence indicated |
|  |  |  | Inclusion of quotes / notes |
|  | Information on screening colonoscopy (66) | Colonoscopy preparation (7) | Reported: yes / no |
|  |  | Colonoscopy sedation (4) | Correct: yes / no / unclear |
|  |  | Procedure (13) | Presentation format: text / number / chart / Table / figure |
|  |  | Test characteristics (7) |  |
|  |  | Conduct in response to test results (3) | Evidence level reported: yes / no / lack of evidence indicated |
|  |  | Benefit (disease-specific incidence and total mortality) (9) | Inclusion of quotes / notes |
|  |  | Risks and adverse effects including overdiagnosis (23) |  |
|  | Information on FOBT (32) | Procedure (9) | Reported: yes / no |
|  |  | Test characteristics (8) | Correct: yes / no / unclear |
|  |  | Conduct in response to test results (3) | Presentation format: text / number / chart / Table / figure |
|  |  | Benefit (disease-specific incidence and total mortality) (9) |  |
|  |  |  | Evidence level reported: yes / no / indication of lack of evidence |
|  |  | Risks and adverse effects including overdiagnosis (3) |  |
|  |  |  | Inclusion of quotes / notes |
| **Generic** |  |  |  |
| **B. Formal issues (33)** | Formal issues (33) | Author and stakeholders involved (14) | Reported: yes / no |
|  |  | Editorial independence (6) | Inclusion of quotes / notes |
|  |  | Sources and currentness of data (8) |  |
|  |  | Aim and target group (5) |  |
| **C. Presentation** & **understandability (59)** | Readability / comprehensibility (29) | Language (18) | Present: yes / mostly yes / mostly no / no / not applicable |
|  |  | Sentences (4) |  |
|  |  | Content structure (3) | Inclusion of quotes / notes |
|  |  | Numerical data (4) |  |
|  | Layout (30) | Structure (11) | Present: yes / mostly yes / mostly no / no / not applicable |
|  |  | Writing/font (6) |  |
|  |  | Visual elements (9) | Inclusion of quotes / notes |
|  |  | Design (4) |  |
| **D. Neutrality** & **balance (7)** | Neutrality and balance (7) | Calls for participation (1) | Present: yes / no / unclear |
|  |  | Fear / downplay (4) | Inclusion of quotes / notes |
|  |  | Uneven presentation of procedures (2) |  |

* One comprehensive criterion on the overall correctness of the information is not shown.

FOBT: faecal occult blood test
