# Supplementary material for: Communicating the Benefits and Harms of Colorectal Cancer Screening Needed for an Informed Choice: A Systematic Evaluation of Leaflets and Booklets
Source: PLoS One. 2014 Sep 12;9(9):e107575. doi: 10.1371/journal.pone.0107575 (PMC4162645; doi:10.1371/journal.pone.0107575)
Supplement: Table S2 — (DOC) [file pone.0107575.s002.doc]

Table S2: CRC screening leaflets (n=28) and booklets (n=13) included in the analysis

| **L/B** | **Editor / Year of publication (if available)** | **Translated title (German title)** |
| --- | --- | --- |
| B | Professional Association of Office-based Gastroenterologists Germany | I’ll go … I’ll go to the colorectal cancer screening  (Ich geh' da hin…ich geh zur Darmkrebsvorsorge) |
| B | German Cancer Society 2005 | Preventing colorectal cancer - Questions and answers (Darmkrebs verhindern - Fragen und Antworten) |
| B | German Cancer Society 2006 | Don’t give cancer a chance (Dem Krebs keine Chance) |
| B | German Cancer Society 2009 | Patient guide to colorectal cancer (Patientenratgeber Darmkrebs) |
| B | German Cancer Aid 2009 | Colorectal cancer - The Blue Guides (Darmkrebs - Die Blauen Ratgeber) |
| B | Falk Foundation 2009 | Colorectal cancer diseases (Darmkrebserkrankungen) |
| B | Gastro League | Colorectal cancer (Dickdarmkrebs) |
| B | Regional Association of SHI Accredited Physicians of Bavaria 2009 | Patient information - Early diagnosis of colorectal cancer (Patienteninformation Darmkrebsfrüherkennung) |
| B | Cancer Society of North Rhine-Westphalia 2009 | Colorectal Cancer, Prevention - Treatment - Aftercare (Dickdarmkrebs, Vorbeugung - Behandlung - Nachsorge) |
| B | Lower Saxon Cancer Society 2007 | Cancer. Prevention, early diagnosis, warning signs (Krebs. Vorbeugen, Früherkennung, Warnzeichen) |
| B | Technicians Health Insurance 2009 | Early detection of colorectal cancer (Darmkrebsfrüherkennung) |
| B | University of Hamburg 2011 | Early detection of colorectal cancer (Darmkrebs Früherkennung) |
| B | Knowledge Network evidence.de of the University Witten/Herdecke GmbH 2005 | Patient guideline for prevention and early diagnosis of colorectal cancer (Patientenleitlinie Vorbeugung und Früherkennen von Dickdarmkrebs) |
| L | Local Social Health Insurance 2002 | Preventing colorectal cancer (Darmkrebs vorbeugen) |
| L | Baden-Wuertemberg against Colon Cancer 2010/2011 | Appearances can be deceptive (Der schöne Schein kann trügen) |
| L | Bavarian State Ministry for Environment, Health and Consumer Protection 2007 | Bavaria against colorectal cancer (Bayern gegen Darmkrebs) |
| L | Authority for Social Policy, Familiy, Health and Consumer Protection | Hamburg against colorectal cancer (Hamburg gegen Darmkrebs) |
| L | German Cancer Aid 2010 | Colorectal cancer detection - Information. Gathering consideration. Decision-making.(Darmkrebs erkennen - Informieren. Nachdenken. Entscheiden.) |
| L | German Cancer Aid | Through thick and thin (Durch dick & dünn) |
| L | German Cancer Information Service | Early diagnosis of colorectal cancer - What should I know? (Darmkrebsfrüherkennung - Was sollte ich wissen?) |
| L | Germany against Colon Cancer (dbp Communication GmbH & Co. KG) | Early diagnosis of colorectal cancer saves lives (Darmkrebs-Vorsorge rettet Leben) |
| L | Felix Burda Foundation | 11 steps through colonoscopy - simply simple! (In 11 Schritten durch die Darmspiegelung - einfach einfach!) |
| L | Gastro League | Fighting colorectal cancer (Kampf dem Darmkrebs) |
| L | Federal Committee of Physicians and Health Insurance Funds 2003 | Leaflet on early diagnosis of colorectal cancer (Merkblatt zur Darmkrebsfrüherkennung) |
| L | Berlin Association of SHIAccredited Physicians | Colorectal cancer is the most common cancer (Darmkrebs ist der häufigste Tumor) |
| L | Regional Working Group Onkology Physicians Care Brandenburg | Drop your trousers … Why not? (…warum nicht mal die Hose runterlassen?) |
| L | Regional Working Group Onkology Physicians Care Brandenburg | So it will be long before your last breath. (Damit die Puste lange hält) |
| L | Regional Working Group Onkology Physicians Care Brandenburg | Participation is vital! (Ein lebenswichtiger Einsatz!) |
| L | Regional Working Group Onkology Physicians Care Brandenburg | So that grandpa will be around a long time (Damit Opa noch lange hält) |
| L | Regional Working Group Onkology Physicians Care Brandenburg | A matter of vital importance (Eine lebenswichtige Amtshandlung) |
| L | Regional Working Group Onkology Physicians Care Brandenburg | Life is nothing without me! (Ohne mich läuft hier gar nichts!) |
| L | Regional Working Group Onkology Physicians Care Brandenburg | Because I prefer to look at the radishes from above! (Weil ich die Radieschen lieber von oben betrachte!) |
| L | Saarland Ministry for Health and Consumer Protection | Preventing colorectal cancer - for life's sake (Darmkrebs Verhindern - dem Leben zuliebe) |
| L | Saarland Ministry for Health and Consumer Protection | I feel good (I feel good) |
| L | Saarland Ministry for Health and Consumer Protection | Participation - early diagnosis - Saving lives (Mitmachen - Früh erkennen - Leben retten) |
| L | Network against Colon Cancer | When it's about colorectal cancer, I get serious (Wenn es um Darmkrebs geht, hört bei mir der Spass auf) |
| L | LebensBlicke Foundation | A moment for your life! (Ein Augenblick für Ihr Leben!) |
| L | LebensBlicke Foundation | Self-test: determine your personal risk of colorectal cancer (Selbsttest zu Ihrem persönlichen Darmkrebsrisiko) |
| L | LebensBlicke Foundation | Take action against colorectal cancer and its precursors (Aktiv gegen Darmkrebs und seine Vorstufen) |
| L | LebensBlicke Foundation | Prevention instead of suffering: take action against colorectal cancer (Vermeiden statt Leiden: Aktiv gegen Darmkrebs) |
| L | LebensBlicke Foundation | Saving a 1000 lives (1000 Leben retten) |

F: Leaflet, B: Booklet
